# Drug Literacy in Iran: the Experience of Using “The Single Item Health Literacy Screening (SILS) Tool” 

**Published:** 2014

**Authors:** Farzad Peiravian, Hamid Reza Rasekh, Hasan Jahani Hashemi, Navid Mohammadi, Nahid Jafari, Kianoosh Fardi

**Affiliations:** a *Department of Phamacoeconomics and Pharmaceutical Management, School of Pharmacy, Shahid Beheshti University of Medical Sciences, Tehran, Iran.*; b *Department of Biostatistics, School of Medicine, Qazvin University of Medical Sciences, Qazvin, Iran.*; c *Department of Community Medicine, Faculty of Medicine, Tehran University of Medical Sciences, Tehran, Iran.*; d *Health Management Network Center, Ministry of Health and Medical Education, Iran. *; e *Deputy of Food and Drug, Shahid Beheshti University of Medical Sciences, Tehran, Iran. *

**Keywords:** Drug literacy, Screening tools, Educational status, Health education, Assessment tools

## Abstract

Drug and health literacy is a key determinant of health outcomes. There are several tools to assess drug and health literacy. The objective of this article is to determine drug literacy level and its relationships with other factors using a single item screening tool. A cross-sectional survey was conducted among 1104 people in Qazvin province, Iran. Based on the proportional-to-size method, participants over 15 years old with ability to read were recruited randomly from 6 counties in Qazvin province and were interviewed directly. To determine drug literacy relationship with other variables, Chi-Square and t-test were used. Also, logistic regression model was used to adjust the relationship between drug literacy and other relevant variables. Response rate in clusters was 100%. Findings showed that inadequate drug literacy in Qazvin province is 30.3% and it was in association with (1) age (p = .000), (2) marital status (p = .000), (3) educational attainment (p = .000), (4) home county (p = .000), (5) residing area (p = .000), (6) type of basic health insurance (p = .000), (7) complementary health insurance status (p = .000), and (8) family socioeconomic status (p = .000). After adjusting for these variables using logistic regression model, the association between (1), (3), (4), (5) and (8) with drug literacy level was confirmed. The analysis also showed that this method can also be used in other health care settings in Iran for drug and health literacy rapid assessment.

## Introduction

The “health literacy” term was first used in the 1970s . According to The Patient Protection and Affordable Care Act, health literacy is defined as “the degree of capacity of individuals to obtain, process, and understand basic health information and services needed to make appropriate health decisions”(1,2,3). Obviousely, besed on health literacy definition, drug literacy could be defined as a subgroup of health literacy. 

There are some disagreements between researchers about the differences between literacy and health literacy and scope of each of them. Some researchers believe that health literacy is literacy in the health area, while some others think that health literacy and literacy are separate (4).

Many studies have shown the prevalence of limited health literacy in countries (5). A study in the United States found that 14% of the 19,000 participants had below basic, 22% had basic, over half (53%) had intermediate, and 12% had proficient health literacy (6).

Evidences have shown the relationships between health literacy and health status; amount and pattern of use of health services; knowledge and ability to manage chronic diseases and adherence to treatment and medication use (7). It is important to know that even after adjusting for some demographic variables such as age, gender, race, and socio-economic status, the relationships still hold (8, 9, 10 and 11) and are identified well (12).

Moreover, evidence has shown the relationship between inadequate health literacy and utilization pattern of health care services such as higher use of emergency health care services, lower use of preventive services, difficulties with medication dosages and understanding health messages (13,14), reporting illness and perceptions of illnesses and diseases (15).

In medication field, 20- 50% of patients do not take their medication as their physicians have prescribed, which might lead to medication nonadherence. Inadequate health literacy had been considered as one of the most important key factors for medication nonadherence (16). More than half of patients with inadequate health literacy could not understand medicines instruction correctly (17). Misinterpretation of prescription drug labels is doubled in patient with low literacy (8).

On the other hand, individuals with adequate health literacy can use their skills of reading, numeracy, and writing, for health-related subjects within the health-care settings (18). 

Strong link between education and health has led researchers to focus on reading skills and health related outcomes like health knowledge, medication adherence, and hospitalization rates (19, 20).

There are many tools to assess health literacy (8, 21, 22, 23, 24, 25, 26, 27, 28, 29, 30 and 31) The main categories of these tools are:

1- Word recognition tests: Wide Range Achievement Test–Revised (WRAT-R), Rapid Estimate of Adult Literacy in Medicine–Short Form (REALM-SF), Short Assessment of Health Literacy for Spanish Adults (SAHLSA-50), and the Medical Terminology Achievement Reading Test (MART);

2- Reading comprehension tests, which are mostly used in educational settings;

3- Functional health literacy tests: Test of Functional Health Literacy in Adults (TOFHLA), and the Newest Vital Sign (NVS); and,

4- Informal methods (32). 

Time is a very important component in assessing health literacy in the health care setting. Informal methods are the most common methods to assess health literacy. Usually, they consist of some questions to estimate health literacy level. In comparison with other methods, they need less time to be utilized (33). Chew and colleagues (26). defined 3 questions to assess inadequate health literacy. Those questions were successful in identifying individuals with poor reading ability in comparison with Short Test of Functional Health Literacy in Adults (S-TOFHLA)(25,34) a gold standard health literacy instrument (33).

The 3 Questions were:

- “How confident are you in filling out medical forms by yourself?”,

- “How often do you have someone help you read hospital materials?”, and

- “How often do you have problems in learning about your medical condition because of difficulty in understanding written information?” 

Patients select 1 of 5 responses ranging from 1- Never to 5- Always (26, 32, 35).

Wallace and colleagues suggested that 1 question of these 3 questions might be sufficient for detecting limited and marginal health literacy (23).

Morris and colleagues designed a single question,“How often do you need to have someone help you when you read instructions, pamphlets, or other written material from your doctor or pharmacy?”, to assess poor literacy level. They found that this method can assess limited health literacy moderately well in comparison with TOFHLA as gold standard (33). Jeppsen and colleagues found that the above question is one of the measures that can independently predict limited health literacy (29).

Most studies in assessing health literacy are conducted in English speaking countries and it is essential to assess this subject in non- English countries as well. The purpose of this study is to assess drug literacy level in Qazvin province in Iran. The Single Item Literacy Screener (SILS) method was used to do this study.

## Experimental

*Materials and methods*

This study was a part of a larger project, a survey of “Pattern of Utilization of Drug and Pharmaceutical Services”. Data was collected from a random sample (based on proportional to size method) of 1104 participants from both rural and urban areas in 6 counties (Qazvin, Takestan, Alborz, Abyek, Boeen- Zahra, and Avaj) in Qazvin province during April 7 to June8, 2013. A questionnaire was designed to collect demographic data (county, gender, age, residing area, marital status, education, job, basic and complementary insurance, family size and socioeconomic status) as well a single question for assessing health literacy according to Morris, *et al*., method (33);(“How often do you need to have someone help you when you read instructions, pamphlets, or other written material from your doctor or pharmacy?”) with emphasis on medications. Internal validity of questionnaire with respect to clarity, completeness, relevance, face and scoring fields was confirmed by experts. The Cronbach’s Alpha analysis was used for reliability test of the questionnaire and it was found to be 0.869.

Inclusion criteria were: age over 15 years old, having reading skill (at least 1 formal year education), and lack of any disability that can prevent reading. Data was collected in subsequent interviews with households (up to 5 times) by trained interviewers. If the interview attempts were unsuccessful, substituted random sample was used. Data collection method led to a response rate of 100%.

Response choices of SILS were: 1-Never, 2-Rarely, 3- Sometimes, 4-Often, and 5-Always. Cutoff > 2 was used to identify limited drug literacy screening sensitivity. Scores greater than 2 were considered positive, which meant participant had inadequate drug literacy. To assess socioeconomic status of participants, a questionnaire with 30 items, was used. The data was analyzed using Principle Component Analysis (PCA) method (36, 37, 38 and 39) Then participants were categorized in 3 groups, (weak, moderate, and good), based on their socioeconomic status.

The results were analyzed using SPSS.V.16.0. Descriptive analyses were used to calculate frequencies and Chi-square test was used to examine the relationship between qualitative variables while t-test was used for quantitative variables. To determine the final associations between drug literacy level and effective variables, logistic regression model was used.

## Results

1104 individuals participated in this survey who lived in 6 counties in Qazvin province: (Qazvin (584),Takestan(114), Boeen-Zahra (85), Alborz(217), Abyek(86), and Avaj(18). 47.7% of participants were female.20.7% lived in rural areas. Married participants were 72.6%. 85.6 % had basic health insurance. In addition, 37.3% had complementary health insurance. Job status showed that 43.7% of participants were employed. The maximum family size was 7. The mean age of participants was 34.7 years (SD 12.95). The mean of years of education was 9.8 years (SD 4.02), and participants with education less than high school diploma were 80.4%. 27.8% of participants were in weak socioeconomic status. Overall, 30.3% of participants had inadequate health literacy. Characteristics of the participants and their relationship with drug literacy (using Chi-Square and t-test) are shown in Table 1.

**Table 1:** General characteristics according to health literacy level

**Characteristics (n. of participants)**	**n(%)**	**Adequate health literacy (%)**	**Inadequate health literacy (%)**	p- value*
Age (1104)		770 (69.6)	334 (30.3)	.000
Gender (1104)				.399
	Male 577 (52.3)	396 (68.6)	181 (31.4)	
	Female 527 (47.3)	374 (71.0)	153 (29.0)	
Marital status (1104)				.000
	Single 302 (27.4)	238 (78.8)	64 (21.2)	
	Married 802 (72.6)	532 (66.4)	270 (33.6)	
Educational attainment (1104)				.000
	<Diploma 888 (80.4)	566 (63.7)	322 (36.3)	
	>Diploma 216 (19.6)	204 (94.4)	12 (5.6)	
County (1104)				.000
	Qazvin 584 (52.9)	403 (69.0)	181 (31.0)	
	Takestan 114 (10.3)	93 (81.6)	21 (18.4)	
	Boeen-Zahra 85 (7.7)	62 (72.9)	23 (27.1)	
	Alborz 217 (19.7)	154 (71.0)	63 (29)	
	Abyek 86 (7.8)	54 (60.5)	34 (39.5)	
	Avaj 18 (1.6)	6 (33.3)	12 (66.6)	
Residing area (1104)				.000
	Urban area 876 (79.3)	652 (74.4)	224 (25.6)	
	Rural area 228 (20.7)	118 (51.8)	110 (48.2)	
Family size (426)				.330
	1 5 (1.2)	2 (40.0)	3 (60.0)	
	2 54 (12.7)	43 (79.6)	11 (20.4)	
	3 149 (34.9)	86 (57.7)	63 (42.3)	
	4 156 (36.6)	104 (66.7)	52 (33.3)	
	>=5 62 (14.6)	37 (59.7)	25 (40.3)	
Job status (1104)				.709
	Unemployed 622 (56.3)	431 (69.3)	191 (30.7)	
	Employed 482 (43.7)	339 (70.3)	143 (29.7)	
Basic health insurance status (1104)				.867
	With basic health insurance 945 (85.6)	660 (69.8)	285 (30.2)	
	Without basic health insurance 159 (14.4)	110 (69.2)	49 (30.8)	
Basic health insurance type (943)				.000
	Iran Health 128 (13.6)	102 (79.7)	26 (20.3)	
	Iran Health (Villagers) 145 (15.4)	73 (50.3)	72 (49.7)	
	Social security 609 (64.6)	436 (71.6)	173 (28.4)	
	Armed Forces Medical Services 34 (3.6)	24 (70.6)	10 (29.4)	
	Private and others 27 (2.8)	24 (88.9)	3 (11.1)	
Complementary health insurance status (1104)				.000
	have complementary insurance 693 (62.7)	455 (65.6)	238 (34.4)	
	Not have complementary insurance 411 (37.3)	315 (76.6)	96 (23.4)	
Socioeconomic status (1104)				.000
	Weak 307 (27.8)	172 (56.0)	135 (44.0)	
	Moderate 738 (66.8)	546 (74.0)	192 (26.0)	
	Good 59 (5.4)	52 (88.1)	7 (11.9)	

*Based on Chi-Square/ t-test

To adjust the relationships between relevant variables (p < .05, according to Table 1) and drug literacy, logistic regression method was used. According to Table 2, age, educational attainment, county, residing area, and family socioeconomic status showed significant relationship with drug literacy, while marital status as well as basic and complementary health insurance were ruled out. 

**Table 2:** Final Logistic Regression Model for predicting low health literacy

				95% C.I. for OR*	
Characteristics		Sig.	OR*	Lower	Upper
Age		.000	.456	.328	.633
Marital status	Single		1		
	Married	.066	1.474	.975	2.228
Educational attainment	>Diploma		1		
	<Diploma	.000	5.771	3.023	11.016
County		.000			
	Avaj		1		
	Qazvin	.252	1.926	.627	5.916
	Takestan	.003	6.461	1.893	22.051
	Boeen-Zahra	.016	4.381	1.321	14.527
	Alborz	.206	2.116	.662	6.762
	Abyek	.334	1.831	.537	6.245
Residing area	Rural area		1		
	Urban area	.000	2.245	1.475	3.418
Basic health insurance type		.251			
	Iran Health (Villagers)		1		
	Iran Health	.371	.726	.359	1.465
	Social security	.611	1.153	.668	1.997
	Armed Forces Medical Services	.762	.856	.313	2.338
	Private and others	.085	3.525	.839	14.808
Complementary health insurance status	have complementary insurance 693 (62.7)		1		
	Not have complementary insurance 411 (37.3)	.266	1.232	.853	1.778
Family socioeconomic status		.001			
	Weak		1		
	Moderate	.000	1.891	1.334	2.681
	Good	.072	2.996	.922	6.755

*OR: Odds Ratio

## Discussion

This is the first study to identify drug literacy level and its relationship with other demographic variables using SILS method in Iran. Level of inadequate drug literacy in Qazvin province was found at 30.3%. The main findings (Table 1) showed the correlation between drug literacy and age (p= .000), marital status (p= .000), educational attainment (p= .000), county (p= .000), residing area (p= .000), basic health insurance type (p= .000), complementary health insurance status (p= .000) and socioeconomic status (p= .000). Moreover, those findings showed that there are no correlation between drug literacy and gender, family size, job status and basic health insurance type. According to Table 2, after controlling confounding effect of variables using logistic regression model, findings showed that people were more likely to have inadequate drug literacy if they are older (p= .000, OR= .456; 95% confidence interval (CI): .328- .633), have less educational attainment (< Diploma) (p= .000, OR= -5.771; CI:-3.023- 111.016), reside in rural area (p= .000, OR= -2.245; CI: 1.475- 3.418), living in Avaj county (p= .000) and weaker socioeconomic status (p= .001). 

Today in Iran, elders have less educational attainment, based on which they are expected to have inadequate drug literacy. Their ability to read and comprehend medical and pharmaceutical subjects is less than the others, which explains the negative association between age and drug literacy level. 

People with less formal education are more likely to have difficulties in understand medical and pharmaceutical reading materials. It seems that people who live in rural area and Avaj county, have less educational attainment and that makes them weaker in drug literacy. People in good socioeconomic status are expected to have more opportunities to complete their formal education, which results in higher drug literacy level. Moreover, they are more sensitive about their health conditions and that helps them pay better attention to medical and pharmaceutical matters.

These findings are consistent with the results of some previous studies.Tehrani Banihashemi and his colleagues used TOHFLA method to evaluate health literacy in Qazvin and 4 other provinces in Iran in 2007. They found out that overall health literacy level in those provinces was low (56.6%) in relationship with age, formal education level, and economic status, using logistic regression model analysis (40). Improvments in educational level, socioeconomic status, and community awareness in health might be the causes of the decrease drug illiteracy level in Qazvin province from 56.6% in 2007 to 30.3% in 2013. White, *et al., *found the relationship between health literacy and age and economic status (17). Gazmararian and his colleagues have shown significant correlation between health literacy and age, education level, race, language and job (41). 

In addition, Emmerton, *et al., *stated the relationship between health literacy and age, education, gender, and language (42). Rudd, *et al*., showed the correlation between health literacy and economic status and education level (43). They couldn’t, however, demonstrate its relationship and gender. The relationship between health literacy and education, age, and geographical location was demonstrated in Becker and his colleagues study (30). On the other hand, neither they could found a correlation between health literacy and gender. There are other studies that support the result of this survey (5,8,31 and 44) 

After adjusting the results using logistic regression model, the relationship between drug literacy and marital status, as well as basic and complementary health insurance was not confirmed.

The limitation of this study was the lack of good accompany of some participants, especially from urban areas, mostly in socioeconomic status questions. The study was able to address some of the limitations which have been mentioned in Morris’study (33). 

## Conclusion

The results of this study have shown that the older an individual, the more inadequate his/her drug literacy will be increased. People with less educational level (<Diploma) were 5.7 times more probable to have inadequate drug literacy. Moreover, living in Avaj County is correlated with higher level of inadequate drug literacy in comparison with other counties in Qazvin province. As a result, drug literacy has a positive correlation with living in Abyek, Qazvin, Alborz, Boeen Zahra, and Takestan counties. It seems that living in a rural area increases the chance to have inadequate drug literacy nearly 2.3 times in comparison with living in urban areas. Finally, low socioeconomic status may increase inadequate drug literacy level nearly 3 times more compared to good socioeconomic status.

drug literacy level is an important characteristic which can help people evaluate their own health situation properly. It can improve patients’ medical adherence, decrease their misunderstanding of pharmaceutical dosages, and prevent self-administration of medications.

The authors recommend that the government supports drug literacy promotional programs in all educational levels and other fields of intervention such as primary health care settings, clinics, pharmacies, and hospitals, using all kinds of media.
